# Assessing Herbivore Foraging Behavior with GPS Collars in a Semiarid Grassland

**DOI:** 10.3390/s130303711

**Published:** 2013-03-15

**Authors:** David J. Augustine, Justin D. Derner

**Affiliations:** 1 Rangeland Resources Research Unit, United States Department of Agriculture–Agricultural Research Service, 1701 Centre Avenue, Fort Collins, CO 80525, USA; 2 Rangeland Resources Research Unit, United States Department of Agriculture–Agricultural Research Service, 8408 Hildreth Road, Cheyenne, WY 82009, USA; E-Mail: Justin.Derner@ars.usda.gov

**Keywords:** activity sensors, classification tree, GPS collars, grazing behavior, herbivore distribution, livestock, rangeland, shortgrass steppe, ungulate

## Abstract

Advances in global positioning system (GPS) technology have dramatically enhanced the ability to track and study distributions of free-ranging livestock. Understanding factors controlling the distribution of free-ranging livestock requires the ability to assess when and where they are foraging. For four years (2008–2011), we periodically collected GPS and activity sensor data together with direct observations of collared cattle grazing semiarid rangeland in eastern Colorado. From these data, we developed classification tree models that allowed us to discriminate between grazing and non-grazing activities. We evaluated: (1) which activity sensor measurements from the GPS collars were most valuable in predicting cattle foraging behavior, (2) the accuracy of binary (grazing, non-grazing) activity models *vs.* models with multiple activity categories (grazing, resting, traveling, mixed), and (3) the accuracy of models that are robust across years *vs.* models specific to a given year. A binary classification tree correctly removed 86.5% of the non-grazing locations, while correctly retaining 87.8% of the locations where the animal was grazing, for an overall misclassification rate of 12.9%. A classification tree that separated activity into four different categories yielded a greater misclassification rate of 16.0%. Distance travelled in a 5 minute interval and the proportion of the interval with the sensor indicating a head down position were the two most important variables predicting grazing activity. Fitting annual models of cattle foraging activity did not improve model accuracy compared to a single model based on all four years combined. This suggests that increased sample size was more valuable than accounting for interannual variation in foraging behavior associated with variation in forage production. Our models differ from previous assessments in semiarid rangeland of Israel and mesic pastures in the United States in terms of the value of different activity sensor measurements for identifying grazing activity, suggesting that the use of GPS collars to classify cattle grazing behavior will require calibrations specific to the environment and vegetation being studied.

## 1. Introduction

Advances in the use of global positioning system (GPS) technology to track domestic and wild herbivores have revolutionized the study of ungulate distribution and movement patterns. However, the use of GPS technology to study the foraging behavior of free-ranging herbivores has been limited by the inability to distinguish between foraging locations and locations of other activities such as bedding, resting, or traveling. A recent review of dietary selection by domestic herbivores [1] noted that advances in this subject will require “better linking of behavioral and nutritional studies”, and “more explicit spatial models of foraging behavior that incorporate multiple scales of decision making.” Advances will therefore require not just recording the location of a herbivore over time, but increased use of methods that can determine both where and when herbivores are feeding. Similarly, improved understanding of how herbivores affect vegetation dynamics and ecosystem processes often requires knowing the spatiotemporal pattern of forage utilization within an area, rather than simply the number of herbivores using the area [2–4].

The distance moved by a herbivore over a given time interval is expected to be an indicator of its activity. For example, short distances are likely to indicate stationary behaviors (standing, bedding), long distances to indicate traveling without feeding, and intermediate distances to be associated with feeding. However, for an interval lasting several minutes or more, there can be substantial overlap in distances moved during each of these activities, which is further compounded by error in GPS location data [5,6]. Over intervals of a minute or less, movement rates or distances may be better able to discriminate between resting, feeding and traveling, but require collection of substantially larger datasets and associated increases in equipment battery requirements [7,8]. Many commercially available GPS collars can be configured to include an activity sensor which records movements in a given direction over a particular time period, and the angle of the collar which indicates the orientation of the head of the animal When these activity sensors are used alone, or in combination with distances moved by the animal, it is possible to more accurately discriminate between grazing and non-grazing activities [9,10]. Calibration of GPS collars to predict animal activity, however, requires direct field observations of the animals, which can be labor intensive. Activity calibrations for GPS collars on cattle have been conducted with limited sample sizes [1–3,9,10], but it remains unclear whether a general predictive equation can be developed, or whether the kinds of movements measured by activity sensors are robust across different types of rangelands. In addition, previous studies have not examined the degree to which calibrations are robust with respect to interannual variation in precipitation and forage availability.

We used direct observations of cattle grazing shortgrass steppe in eastern Colorado, USA, to develop models that use data from Lotek GPS collars to discriminate among different behaviors. We conducted calibration tests in four different years that varied widely in precipitation amount and pattern, and during different months within each growing season. We use classification and regression tree (CART) analysis [11] to ask: (1) how robust are models of cattle grazing activity across different types of rangelands; (2) what aspects of activity sensors are most valuable in predicting cattle foraging behavior; (3) what level of accuracy can be achieved for models that are robust across years *vs.* models that are specific to a given year, and (4) what is the accuracy of models that predict multiple categories of cattle activity (grazing, resting, traveling) *vs.* binary models of grazing *vs.* non-grazing activity?

## 2. Experimental Section

### 2.1. Study Site

We evaluated yearling steer behavior while they grazed native shortgrass steppe at the USDA-Agricultural Research Service's Central Plains Experimental Range (CPER) located approximately 12 km northeast of Nunn, Colorado, USA (40°50′N, 104°43′W). Mean annual precipitation is 340 mm, and mean growing season (April-August) precipitation is 241 mm. Topography is characterized by gently undulating plains. Blue grama (*Bouteloua gracilis* (Willd. Ex Kunth) Lag. ex Steud) and buffalograss (*Buchloe dactyloides* (Nutt.) J. T. Columbus) are the dominant grasses (>80% of ANPP) and scarlet globemallow (*Sphaeralcea coccinia* (Nutt) Rydb.) is the most abundant forb; mean annual aboveground plant production is ∼75 g m^−2^ [12].

### 2.2. GPS Collars and Cattle

Steers were fitted with Lotek 3300LR GPS collars (Lotek Engineering, Newmarket, Ontario, Canada; www.lotek.com) that weighed 1.2 kg. Collars included a buckle that allowed us to rapidly place them around the necks of steers and to remove them while the animals were restrained within a chute. Once the collar was on the steer, we examined the fit around the neck, and adjusted the length of the collar belt to ensure that a person's fingers fit between the animal's neck and the inside of the collar. Collars were set to record GPS positions at 5-minute intervals. Collars included a dual axis activity sensor that recorded the number of up-down (Yact) and side-to-side movements (Xact; up to a maximum of 255 detected in each 5 minute interval), corresponding to the same interval as the GPS fixes. In addition to the number of movements along the y-axis, sensor data included the percentage of time that the y-axis sensor was in the “down” position (referred to hereafter as percent of time with head down). See reference [2] for additional details on the activity sensors. Steers were crossbred *Bos taurus* and were studied during the 15 May–30 September grazing season with a new group of steers studied each year; during the grazing season, they increased in live weight from approximately 270 kg to 410 kg.

### 2.3. Observations of Cattle Activity

We concurrently collected GPS collar data and direct observations of 5–9 steers during each of 12 collar deployments during the grazing seasons of 2008 (four deployments during May–September), 2009 (two deployments during May–July), 2010 (four deployments during May–September), and 2011 (two deployments during June–August). Each deployment lasted 21–30 days, during which collars were distributed among three different pastures with three collars per pasture. Deployments occurred in two different sets of pastures (six pastures total) which were 65–390 ha in size. Visual observations of two or three of the collared steers in each pasture were conducted during daylight hours for 1 day of each deployment. Observations lasted an average of 5.1 h per day. Observations began during morning hours and lasted until early afternoon, and were scheduled to encompass bouts of both grazing and resting or bedding within a given day.

Direct observations followed the methods of Ganskopp and Bohnert [7]. One observer was assigned to each steer. Observers used a hand-held GPS device to record time, in order to ensure synchrony with time being recorded by the GPS collars. Cattle activity was classified into categories consisting of grazing (including grazing while walking as long as the animal's head was down and it harvested forage), traveling (walking without grazing), standing, bedding, grooming, drinking, or consuming mineral salts. Activity timing and duration were recorded every 30 seconds. When an animal switched from one activity to another, observers mentally noted the precise transition time on the hand-held GPS unit. If the new activity persisted for >15 seconds, then the transition was recorded. If the animal resumed its prior activity in <15 seconds, then the interlude was ignored. Each 5 minute interval was assigned to the category of “grazing” if the animal was grazing for ≥50% of the 30 second intervals. Those not classified as grazing were then classified as either “traveling” (≥50% of the 30 second intervals spent traveling) or “resting” (≥50% of the 30-second intervals spent bedding, standing, drinking, grooming, and/or consuming salts). Remaining observation periods were assigned a classification of “mixed activity”.

### 2.4. Statistical Analysis

We developed binary models predicting whether or not a given 5-minute interval was classified as grazing based on activity sensor data and the distance moved between GPS fixes. Activity sensor data consisted of total movements recorded along the horizontal axis (Xact), total movements recorded along the vertical axis (Yact), the sum movements in both directions (XYsum), and percent time with the head down. Models were developed using CART analysis [11]. Model splits were determined by maximizing a LogWorth statistic that is related to the likelihood ratio chi-square statistic (*G*^2^) associated with each potential split, where *G*^2^ is defined on the basis of an entropy index (see reference [11] for a discussion of possible indicies for determining splitting criteria). The LogWorth statistic is equal to −log_10_(*p*-value), where the *p*-value for a given split is calculated in a manner accounting for the number of different ways in which splits could occur. Details on how *p*-values are calculated during the fitting of classification trees, and their relationship to the likelihood ratio chi-square statistic, are provided by Sall [13]. All candidate classification tree models were fit using the JMP statistical package, version 8.0. For each deployment, we randomly selected one collar and withheld all of the 5-minute intervals with observation data for that collar from the training data set used to fit models. The set of withheld observations (*n* = 681 5 minute intervals) is referred to as the validation dataset. We selected models by first fitting classification trees of increasing complexity (increasing number of splits) to the training dataset (*n* = 5,367 5 minute intervals), and then selecting the model that minimized misclassification rates for the validation dataset [11]. We also fit models based on the data collected in all 4 years, and compared them with models fit separately to each year of data.

## 3. Results

**Figure 1.**
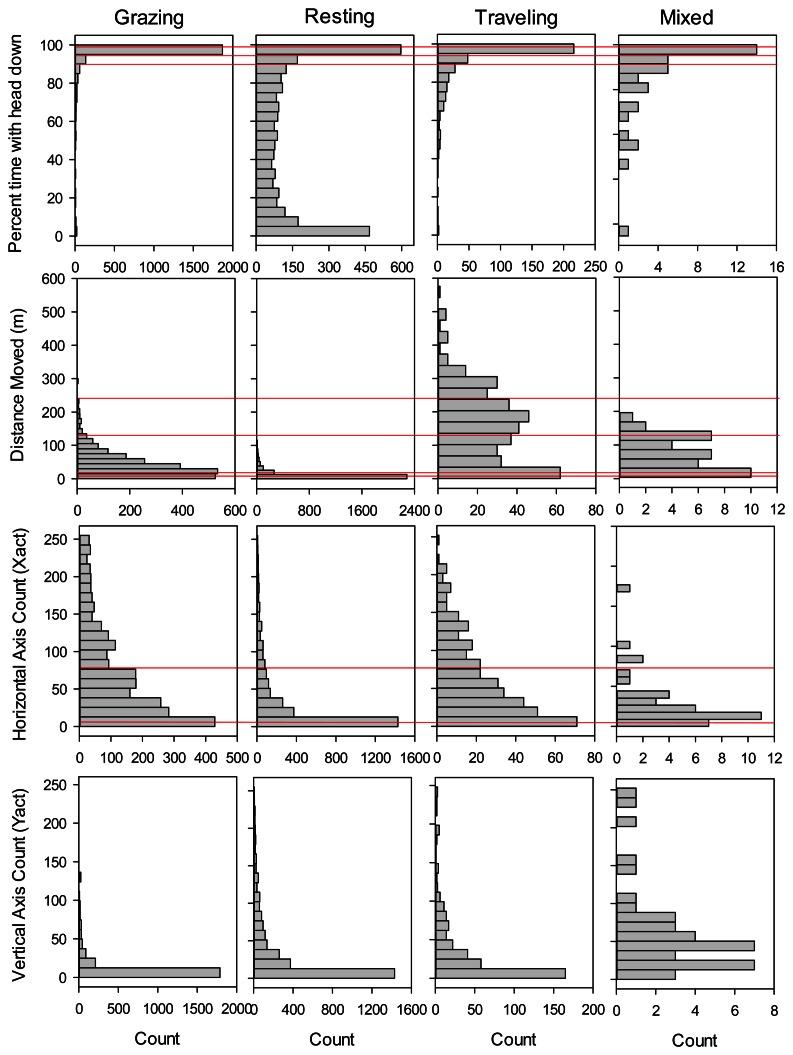
Frequency distributions of movement distances and activity sensor measurements collected at 5 minute intervals using Lotek 3300LR GPS collars on yearling steers in eastern Colorado, for each of four activity categories. The “mixed” category refers to 5 minute intervals where neither grazing, resting, nor traveling occurred for 50% or more of the interval. Red lines show thresholds identified by the final selected classification tree discriminating among the four activity categories (see Figure 3).

In total, we collected concurrent GPS collar and direct observation data for 98 steer-collar combinations and 6,048 5-minute intervals. These measurements spanned years that varied considerably in forage availability, with wet years in 2009 and 2010 (353 and 285 mm growing season precipitation; peak standing crop of 2439 and 1518 kg/ha) and dry years in 2008 and 2011 (240 and 245 mm growing season precipitation; peak standing crop of 531 and 732 kg/ha). We first examined histograms of movement distances and sensor measurements for the training dataset for each of the four activity categories (Figure 1), in order to gain insight to how they may contribute to models differentiating among activities.

Shape of the frequency distributions differed among Grazing, Resting and Traveling most notably in terms of percent of the 5-minute interval in which the Y-axis sensor was in the headdown position (Headdown), and distance moved in the 5-minute interval (DIST). Headdown was bimodal for Resting, and strongly skewed toward 100% for Grazing. DIST was strongly skewed toward zero for Resting, and bimodally distributed for Traveling. In contrast, shape of the frequency distributions for movements in the horizontal and vertical directions was similar for Grazing, Resting and Traveling. In the case of movements in the vertical direction, the vast majority of counts were between 0 and 12 for all three primary activity categories (Figure 1). The ‘mixed’ category comprised only 0.7% of the total number of observations, and generally exhibited more variable frequency distributions than the other activity categories.

**Figure 2.**
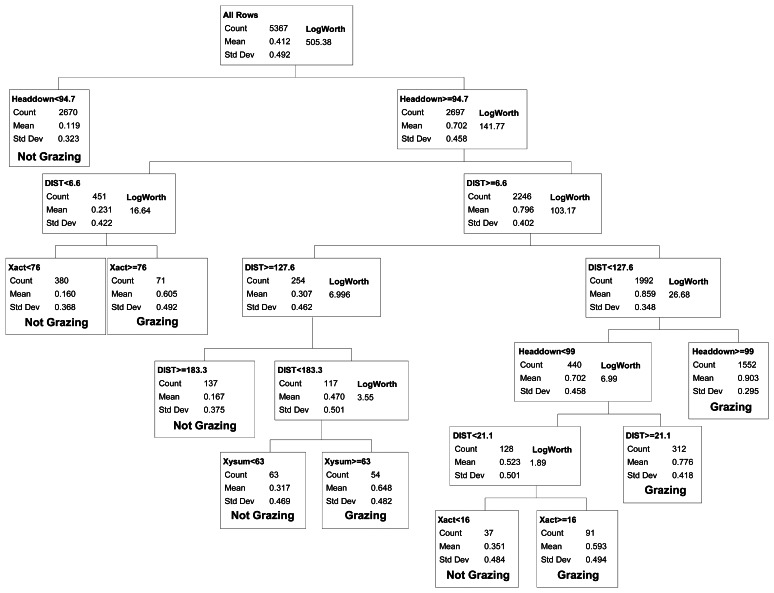
Selected binary classification tree differentiating cattle grazing *vs.* non-grazing activities based upon movement distances and activity sensor data collected at 5-minute intervals with Lotek 3300LR GPS collars on yearling steers in eastern Colorado. Misclassification rates for this model for both the training and validation datasets are shown in bold in Table 1. Number of observations refers to the training dataset. Variables in the classification tree are DIST = Distance in meters between GPS fixes over a 5-minute interval, Xact = number of movements in the horizontal direction (head movement left to right or right to left) detected by the activity sensor, Yact = number of movements in the vertical direction (head movement up and down) detected by the activity sensor, XYsum = Xact + Yact, and Headdown = percent of the 5-minute interval in which the Y-axis sensor was in the head down position. See text for a definition of the LogWorth statistic.

We evaluated binary models that differentiated between grazing *vs.* all other activities (resting, traveling and mixed) combined. Classification tree demonstrated showed that Headdown and DIST were the two most important variables in all models (Figure 2). When observations from all years were modeled collectively, the first model split differentiated between intervals with Headdown < 94.7% (non-grazing) *vs.*≥ 94.7% (grazing; *r*^2^ = 0.35). Three additional splits yielded significant model improvement (*r*^2^ = 0.51) by partitioning the Headdown ≥ 94.7% category into DIST < 6.59 m (non-grazing) and DIST ≥ 6.59 m (grazing), and then partitioning the former group on the basis of motion along the x-axis of the activity sensor (Xact > 76 = grazing), and the latter on the basis of distance travelled (DIST < 127.57 = grazing).

At this point, further splitting yielded much smaller improvements in model fit, and certain splits continued to reduce misclassification rates for the validation data. For the category with Headdown < 94.7% (mean probability of grazing = 0.11), at least 4 additional splits were required to identify one subset with a mean probability of grazing greater than 0.5. This yielded small improvements in model predictive capacity for the training dataset, and either increased error in the validation dataset (four additional splits, yielding an 8-split model) or provided no net improvement in error for the test dataset (five additional splits, yielding a 9-split model; Table 1). These splits were therefore excluded from the final selected model.

For the category with Headdown ≥94.7% and its subsets, additional splits in the classification tree yielded improved model predictive capacity both for the training and validation datasets, up to a minimum misclassification rate for a 9-split model (Table 1). The final selected binary model included five categories defining non-grazing activities, and five categories collectively defining grazing activity (Figure 2). Note that as we considered models of increasing complexity (*i.e.*, increasing number of splits), more than one split was required to separate a group of observations into multiple smaller groups that differed in activity. For example, given the 4-split model, two additional splits (6-split model) were required to partition a group of observations into subgroups which included both grazing and non-grazing categories, such that a 5-split model could not be considered. All possible models with four to 11 splits were evaluated against the validation dataset (Table 1) when selecting the final 9-split model presented in Figure 2.

The final selected binary model had overall misclassification rates of 13.8% and 12.9% for the training and validation datasets, respectively. Analysis of variation in the misclassification rate for each collar-steer combination gave a 95% confidence interval of 11.5–15.1% (training dataset) and 8.5–16.8% (validation dataset) for the selected model's overall misclassification rate. The selected model also had a false positive rate (12.2%) similar to the false negative rate (13.5%).

We tested whether the model based on all four years of collar deployments could be improved by fitting separate models for each year. Based on the same model evaluation process using the yearly validation data, we selected 8-split models for 2008, 2009, and 2011, and a 9-split model for 2010. In three of four years (2008, 2009 and 2010), the cumulative model had greater predictive capacity (misclassification rates 1–4% lower) than did yearly models (Table 2). In contrast, the 2011 yearly model's misclassification rate was lower than the cumulative model (Table 2).

**Figure 3.**
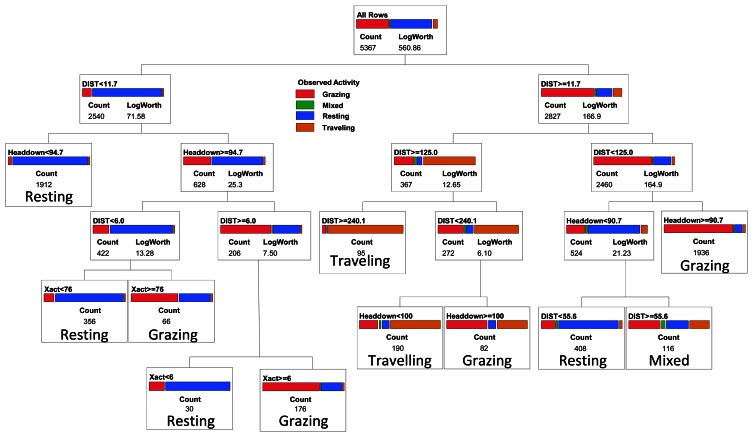
Selected four-category classification tree for cattle activity predicted by movement distances and activity sensor data collected at 5-minute intervals using Lotek 3300LR GPS collars on yearling steers in eastern Colorado. Counts of observations in each box refer to the training dataset used to fit the model. Variables in the classification tree are DIST = Distance in meters between GPS fixes over a 5-minute interval, Xact = number of movements in the X direction detected by the activity sensor, Yact = number of movements in the Y direction detected by the activity sensor, XYsum = Xact + Yact, and Headdown = percent of the 5-minute interval in which the Y-axis sensor was in the head down position. See text for a definition of the LogWorth statistic.

In addition to the binary trees, we evaluated classification trees that partitioned cattle activity into four categories consisting of grazing, resting, traveling, and mixed activity. Following the same process used in selection of the binary classification trees, for the categorical classification tree we selected a 10 split model (Figure 3), which gave a minimum misclassification rate of 16.4% when applied to the validation dataset (112 out of 681 observations misclassified; Table 3). The selected model was most effective in predicting resting activity (11.8% misclassified) and grazing (15.0% misclassified), but less effective in identifying intervals when cattle were traveling (42.5% misclassified), primarily due to inclusion of grazing activity in the category predicted to consist of traveling (Table 3). A small proportion of the intervals in the training and validation datasets (0.7 and 1.2% respectively) were classified as mixed activity because no single activity (grazing, resting or traveling) occurred for more than 50% of the interval; such intervals had no distinctive pattern in terms of movement distance, head position, or number of movements recorded by the activity sensor (Figure 1).

## 4. Discussion

For applications where researchers want to examine cattle foraging distribution, rather than simply the distribution of cattle locations, our binary classification was capable of correctly removing 86.5% of the non-grazing locations from datasets collected by GPS telemetry, while still correctly retaining 87.8% of the locations where the animal was grazing. Our overall misclassification rate (12.9%) was similar to the 12% misclassification rate reported from analyses based on 11 cattle-collar trials in semiarid Israeli rangeland [2], and greater than the 8.3% misclassification rate reported from analyses based on five cattle-collar trials in mesic pastures in Kentucky [3]. Surprisingly, even though these three studies collected data using the same 5-minute GPS fix schedule and a 2-axis activity sensor, they differed considerably in the way the activity sensor measurements were used to classify cattle grazing activity. In Kentucky, only the sum of the horizontal and vertical activity sensor counts was needed to distinguish grazing from non-grazing activity [3]. The Israeli model [2] relied on both horizontal and vertical activity sensor counts plus the distance moved in the 5-minute interval; this pattern was further supported by a second follow-up study in more heterogeneous Israeli rangeland [1]. In contrast, we found that for cattle grazing in semiarid, shortgrass rangeland of Colorado, movement recorded in horizontal and vertical directions provided only limited information in discriminating grazing from other activities (Figure 2). Our model primarily partitioned activity on the basis of the proportion of the 5 minute interval that the vertical sensor was in the head down position and the distance moved in the 5 minute interval. While horizontal activity counts and the sum of horizontal and vertical activity counts were included in our final selected model, they explained substantially less variation than movement distance and time with head down. This difference suggests that foraging behavior and/or the foraging environment of the cattle varied considerably among the three studies. The steers we studied were grazing a short, relatively homogenous sward where animals could repeatedly prehend and ingest bites with minimal head movement. During our observations of the steers, we noted that they rarely raised their heads during bouts of grazing to search for the next bite, thus limiting back-and-forth movements in the horizontal and vertical directions. In particular, movements detected on the vertical axis (Y-act) were frequently less than 12 both when cattle were grazing and resting (Figure 1). In contrast, previous studies examined activity sensors on cattle grazing swards with greater heterogeneity in vegetation structure, either associated with a more productive plant community [2], or with greater variation in height and spacing of the dominant bunchgrasses [1,3]. As a result, we suggest that the use of GPS collars and activity sensors to classify cattle grazing behavior will require calibrations specific to the type of vegetation being studied.

Variation in how GPS receivers are attached to animals is likely to be another important source of error in predicting foraging activity [3,10,14]. Because we developed our classification tree models with a dataset that was independent of the validation dataset (*i.e.*, the validation dataset consisted of collar-steer fittings that were not part of the model training dataset) our model misclassification rates include error due to variation in collar fit. It is still possible that variation among studies in collar fitting methods could contribute to differences in the way that activity sensor measurements predict cattle foraging activity. However, our study used the same type of collars and attempted to place them on the animals in a similar manner as Ungar and colleagues [2,3]. During our extensive field observations of cattle foraging behavior, we consistently noticed a lack of back-and-forth head movements in either the vertical or horizontal directions while animals were grazing in this shortgrass rangeland. We suggest this may explain why the Xact and Yact measurements from the activity sensors did not play a major role in discriminating between grazing and non-grazing activity.

Fitting annual models to predict cattle grazing activity did not improve model accuracy compared to a single model based on all 4 years of measurements combined. This result indicates that increased sample size was more valuable in enhancing model predictive capacity than was the development of annual models to remove the potential influence of interannual variation in cattle foraging behavior. This result also suggests that the wide variation among years in forage biomass did not have a major effect on the movement distances of steers (at least at a 5-minute temporal scale) or their head movement patterns during grazing.

When we separated the non-grazing group into additional categories consisting of resting, traveling and mixed activity, model predictive capacity declined (16.4% misclassification rate) primarily because we could not distinguish the mixed category (no single dominant activity within a 5-minute interval) from either resting or traveling (Figure 2). The ‘mixed’ category is a consequence of the 5-minute recording interval used by the activity sensor and separating the GPS fixes. Inclusion of the mixed category in our model reflects how the use of a 5-minute interval, as opposed to an instantaneous point in time, contributes to classification error. If researchers wish to examine the amount of time that an animal spends grazing each day, then a model that incorporates the full range of possible activities, including 5-minute intervals in which no single activity occurs for the majority of that time period, needs to be considered. Our classification tree placed 2% of the intervals into the “mixed” category where the combination of movement distances and activity sensor measures was not indicative of any distinct activity. By using a shorter time interval (e.g., 1 minute or less; reference [10]) this source of error could likely be reduced further. Conversely, this error will increase with the use of intervals longer than 5 minutes, and may reduce one's ability to effectively assess daily or sub-daily foraging time. For those intervals classified as mixed using our 5-minute interval method, 40% of the time was spent grazing; ignoring the mixed category (*i.e.*, viewing it as a non-grazing category) would only underestimate total daily grazing time by 0.8%.

Other primary sources of error in our classification tree included: (1) animals traveling with their head down, which could be mistakenly classified as grazing; (2) animals standing with their head down, which could be mistakenly classified as grazing, and (3) animals grazing in a small patch with small net movement distance, which could be mistakenly classified as resting. The model did effectively distinguish between resting and traveling, primarily on the basis of the distance travelled with a 5-minute interval. For some applications, this may provide a more valuable classification of cattle activity than a binary model that only discriminates grazing *vs.* non-grazing activity.

## 5. Conclusions

Our findings combined with those of previous studies [1–3] show that GPS collars with dual-axis activity sensors can be used to study the spatial foraging behavior of livestock with reasonable accuracy. By applying such calibrated classification tree models to datasets collected with GPS collars, researchers can more effectively understand factors influencing the foraging distribution of free-ranging livestock in a manner that is not biased by the selection of favored resting or bedding locations [7]. When using such an approach, we recommend developing classification trees specific to the study site based upon direct observations distributed across a range of animals, collars, and forage conditions that are representative of the dataset to which they will be applied. Findings also indicate that use of GPS locations collected at 5 minute or longer intervals is unlikely to classify grazing *vs.* non-grazing activity with error rates lower than 8 to 13%, and unlikely to predict multiple activity categories with error rates lower than 12 to 16%. One study tested combining a tri-axial accelerometer with GPS collars to predict grazing behavior of goats, and found similar error rates of 10–25% [15]. Another study considered how well grazing, resting and travelling behavior of cattle could be predicted using GPS data collected at 10-second intervals and analyzed over 3-minute intervals, without the aid of activity sensors [9]. They formally tested classification accuracy using an independent validation dataset, just as we did, but found a higher misclassification rate of 28.8% [9]. For applications requiring misclassification rates lower than the 13% that we achieved, combining GPS collars with other types of sensors or increased temporal resolution of GPS fixes may be necessary. By using GPS collars in combination with pedometers [2], error rates for a multiple category model can be reduced to 10%. Increasing accuracy further may require combining GPS collars with more specialized and data-intensive approaches such as acoustic sensors [16,17], or the collection and analysis of GPS locations over time periods of a minute or less [10].

## Figures and Tables

**Table 1.** Misclassification rates for binary classification trees differentiating between cattle grazing and non-grazing activities based upon synchronized GPS collar and direct observation data collected for yearling steers grazing shortgrass steppe in eastern Colorado during 2008–2011. The final selected model is shown in bold, and presented in Figure 2.

**Number of Model Splits**	***r*^2^**	**Misclassification Rate**					

		**Training Data**			**Validation Data**		
	
		**Overall**	**False**		**Overall**	**False**	
	
			**Negatives**	**Positives**		**Negatives**	**Positives**
4	0.505	14.3	13.8	15.0	14.1	15.4	12.2
Splitting within the (Headdown ≤ 94.7 %) category							
8	0.549	13.6	12.1	15.9	14.7	14.7	14.7
9	0.551	13.6	12.4	15.3	14.1	15.4	12.2
Splitting within the (Headdown ≥ 94.7 %) category							
6	0.512	14.0	13.0	15.5	13.4	13.6	13.0
**9**	**0.529**	**13.8**	**13.2**	**14.6**	**12.9**	**13.5**	**12.2**
11	0.530	13.7	13.4	14.1	13.2	14.0	12.2

**Table 2.** Misclassification rates (% of total observations) for the validation dataset used to test the binary classification trees predicting cattle activity (grazing *vs.* not grazing) for all 4 years of data combined (cumulative model) and for each year modeled separately.

**Models**	**All Years**		**2008**	**2009**	**2010**	**2011**
**Cumulative**						
Overall Rate	12.9		11.1	9.7	16.8	13.5
False Positive Rate	13.5		15.5	8.5	7.8	12.5
False Negative Rate	12.2		6.5	10.5	20.9	14.6
**Yearly**						
Overall Rate			15.0	12.3	17.7	12.6
False Positive Rate			17.9	9.1	5.1	12.3
False Negative Rate			12.3	14.3	22.8	13.0

**Table 3.** Frequency of observed *vs.* predicted cattle activities based on the selected 10-split categorical classification tree model (Figure 3) applied to the validation dataset.

**Observed**	**Predicted Activity**			
Activity	Grazing	Resting	Traveling	Mixed
Grazing	259	36	16	1
Resting	33	282	4	8
Traveling	6	0	27	3
Mixed	3	2	0	1
